# Supplementary material for: Harmonic to anharmonic tuning of moiré potential leading to unconventional Stark effect and giant dipolar repulsion in WS2/WSe2 heterobilayer
Source: Nat Commun. 2023 Aug 4;14:4679. doi: 10.1038/s41467-023-40329-3 (PMC10403536; doi:10.1038/s41467-023-40329-3)
Supplement: Supplementary file 1 — Supplementary Information [file 41467_2023_40329_MOESM1_ESM.pdf]

Supplementary Information for: Harmonic to anharmonic tuning of moiré potential leading to unconventional Stark effect and giant dipolar repulsion in WS_2/WSe_2 heterobilayer

Suman Chatterjee[1,||], Medha Dandu[1,2,||], Pushkar Dasika[1,||], Rabindra Biswas[1], Sarthak Das[1,3], Kenji Watanabe[4], Takashi Taniguchi[5], Varun Raghunathan[1], and Kausik Majumdar[1*]

[1]Department of Electrical Communication Engineering, Indian Institute of Science, Bangalore 560012, India

[2]Currently with Molecular Foundry, Lawrence Berkeley National Laboratory, Berkeley, CA 94720, United States

[3]Currently with Institute of Materials Research and Engineering (IMRE), Agency for Science, Technology and Research (A*STAR), Singapore 138634, Republic of Singapore

[4]Research Center for Functional Materials, National Institute for Materials Science, 1-1 Namiki, Tsukuba 305-044, Japan

[5]International Center for Materials Nanoarchitectonics, National Institute for Materials Science, 1-1 Namiki, Tsukuba 305-044, Japan

[||]These authors contributed equally

[*]Corresponding author, email: kausikm@iisc.ac.in

Supplementary Note 1:

Polarization dependent SHG measurement technique: A SHG microscopy setup is used to measure the twist angle between the WS_2 and WSe_2 layers. The laser setup consists of a fixed-wavelength femtosecond pulsed laser source (Fidelity HP) centered at 1040 nm with a pulse width of 140 fs and a repetition rate of 80 MHz, which pumps an optical parametric oscillator (Levante-IR) to generate a signal source at 1600 nm wavelength. The signal is fed to a Harmonic generator (HarmoniXX - APE) to produce a harmonic wavelength at 800 nm, which serves as the input laser source for the polarization dependent SHG measurement.

The hetero-bilayer sample is placed on a custom-built rotational mount in an inverted position on the x-y stage of an inverted optical microscope (Olympus IX73). The 800 nm harmonic input laser source is then focused onto the sample using a $\times$ 20 (numerical aperture of 0.75) objective, and the backward second harmonic signal centred at 400 nm was collected using the same objective. The emitted SHG signal is then spatially separated from the input laser source using a dichroic filter and detected using a photomultiplier tube. Bandpass (FWHM of 40 nm) and short-pass filters (cut in wavelength 550 nm) are mounted before the photomultiplier tube to reject any residual pump source and minimize background signal.

For twist angle SHG measurement, the sample is rotated from $0°$ to $180°$ using the rotational mount. For each rotation, a pair of galvo mirrors are used to scan the input laser source over the flake point-by-point in a raster pattern over a field of view of 100 μm $\times$ 100 μm to acquire a two-dimensional SHG intensity map. A 256 $\times$ 256 pixels SHG image is obtained by mapping the PMT signal in every pixel with a resolution of $\approx$ 396 nm. An analyzer is placed in front of the PMT to selectively detect the horizontal component of the SHG signal from the sample with respect to the laboratory horizontal axis.

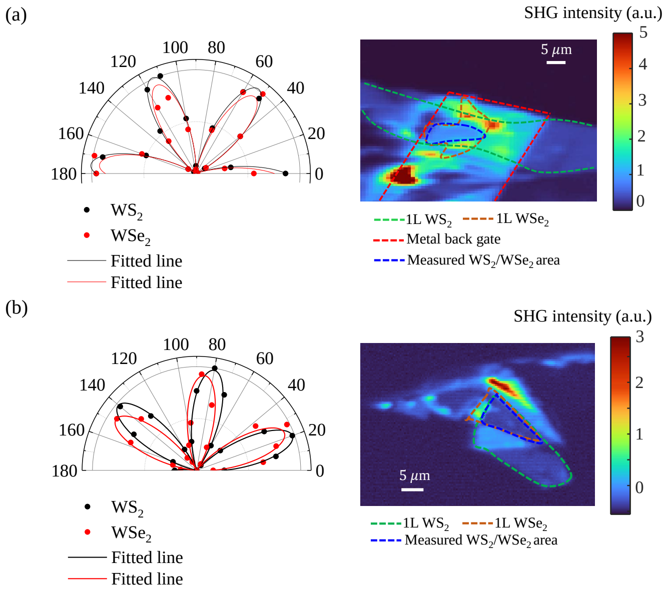

Figure 1: **Polarization-resolved SHG result from the twisted WS_2/WSe_2 stacks.** (a) Polarization resolved SHG polar plot from isolated WS_2 and WSe_2 monolayers is represented for sample D1 in the left panel. Right panel shows SHG map of the full sample (D1), where the isolated 1L WS_2 (green), WSe_2 (orange), and the measured heterojuction area (blue) are marked by dashed lines. From left panel the twist angle is confirmed as $\sim 1°$. The junction area in the the SHG color plot (right panel) shows a drop in intensity, confirming the twist is around AB stacking (60°). (b) Same as (a), but for sample D2 confirming the twist angle to be $\sim 6°$ around AB stacking.

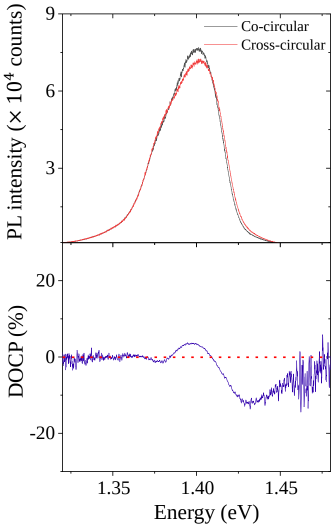

Figure 2: **Circular polarization resolved interlayer excitonic emission.** Circular polarization resolved interlayer excitonic emission for sample D1, with 705 nm (close to WSe_2 intralyer excitonic resonance) excitation. The top and bottom panels show the circular polarization resolved spectra and the degree of circular polarization (DOCP). The alternating sign of the DOCP for different ILE peaks indicates their moiré excitonic nature.

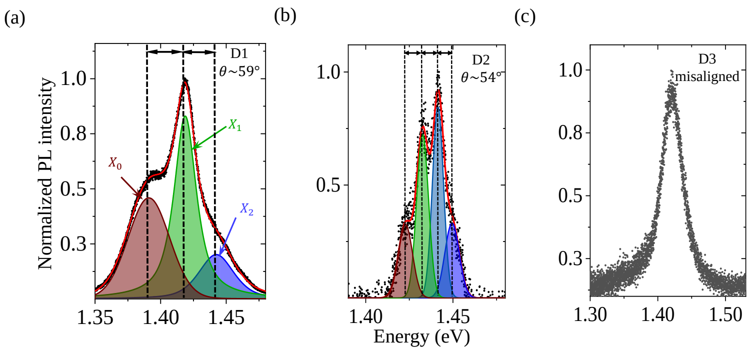

Figure 3: **Twist angle dependence of the moiré exciton emission spectra.** In (a), (b), and (c), the interlayer excitonic PL emission from D1, D2 and D3 is plotted with 532 nm excitation at 4 K. The separation between different ILE peaks reduces from $\sim$ 25 meV (in D1 with a twist angle of 59°) to $\sim$ 9 meV (in D2 with a twist angle of 54°). The largely misaligned sample D3 shows a single broad peak.

Supplementary Note 2:

Moiré potential as two-dimensional harmonic potential well: The depth of the moiré potential is formulated from the spectral separation of the interlayer excitonic emission peaks (Figure 1e, main text). The emission energies are at $\approx$ 25 meV separation from each other (1.392, 1.418 and 1.442 eV for X_0, X_1, and X_2 respectively). This near equal separation suggests an assumption of 2D harmonic oscillator well for moiré pockets is valid [1–4], with energy separation of $\hbar\omega$ (25 meV) between each state. The ground state, X_0, must be then at an energy $\hbar\omega$ above the bottom of the well, and this gives rise to a total well depth of $3\hbar\omega = 75$ meV.

The Hamiltonian for interlayer excitons in WS_2/WSe_2 can be written as [1,3]:

$$H = \hbar\omega' + \frac{\hbar^2 Q^2}{2M} + \Delta(r) \tag{1}$$

Where $\hbar\omega'$ and $\frac{\hbar^2 Q^2}{2m}$ are the constant and kinetic energy of ILE in the center of mass momenta space. M is the effective mass. Here, $\Delta(r)$ is the periodic moiré potential in real space and it can be projected in two dimensions as: $\Delta(r) = \sum_{n=1}^{6} V_n e^{i\vec{\mathbf{b_n}}.\vec{\mathbf{r}}}$, where V_n and $\vec{\mathbf{b_n}}$ are moiré potential and reciprocal lattice vectors, respectively. $\vec{\mathbf{b_n}}$ vectors are calculated from the monolayer lattice vectors, considering 4% lattice mismatch. The 75 meV well depth corresponds to a V_n of $\approx$ 21 meV. Thus $\Delta(r)$ is obtained and plotted in Figure 2c (main text).

Supplementary Note 3:

Formulation of 1D Poisson equation: To understand the electrostatics of the WS_2/WSe_2 moiré heterojunction, we numerically solve a 2D Poisson equation $[\nabla^2\phi = \sigma\delta(z)]$ where WS_2/WSe_2 heterobilayer is considered to be a 2D sheet of charge at $z = 0$ plane with a 2D charge density $\sigma = q(p - n + N)$. Here p, n are the 2D density of electron and hole, respectively. N refers to the unintentional doping in the heterobilayer at zero gate voltage. Under positive gate voltage, $p \approx 0$, and

$$n(x) = N_{2D} log[1 + exp(\frac{E_F - E_C(x) + q\phi(x)}{k_B T})] \tag{2}$$

where N_{2D} is the 2D density of states. E_F is the Fermi level and is referenced as zero. Both conduction (CB) and valence band (VB) profiles are spatially varying according to the spatial bandgap modulation in a moiré superlattice. Only CB (WS_2) and VB (WSe_2) profiles at K point $[E_C^K(x), E_V^K(x)]$ corresponding to the direct bandgap are considered. The moiré potential for electrons and holes is assumed to be a simple cosine profile as: $E_C^K(x) = E_{C0}^K(x) + \Delta E_G^K cos(k_x x)$ and $E_V^K(x) = E_{V0}^K(x) - \Delta E_G^K cos(k_x x)$ with a spatially varying bandgap of $E_G^K = (E_{C0}^K - E_{V0}^K) + 2\Delta E_G^K cos(k_x x)$. Here $k_x = 2\pi/a_M$, $a_M = 7.3$ nm is the moiré period, and $\Delta E_G^K = 75$ meV.

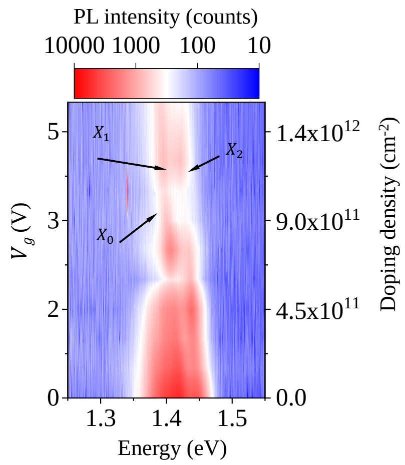

Figure 4: **Gate dependent color plot showing doping density.** With increasing V_g, the n-doping density numbers are shown in the right axis. The highest doping density achieved is 1.5×10^{12} cm^{-2} at $V_g = 5$V. The doping density is estimated assuming charge neutral voltage as 0 V, and by only taking into account the geometrical capacitance of the hBN dielectric, ignoring any channel capacitance effect which comes in series with the gate dielectric capacitance. Hence the quoted doping density is likely overestimated.

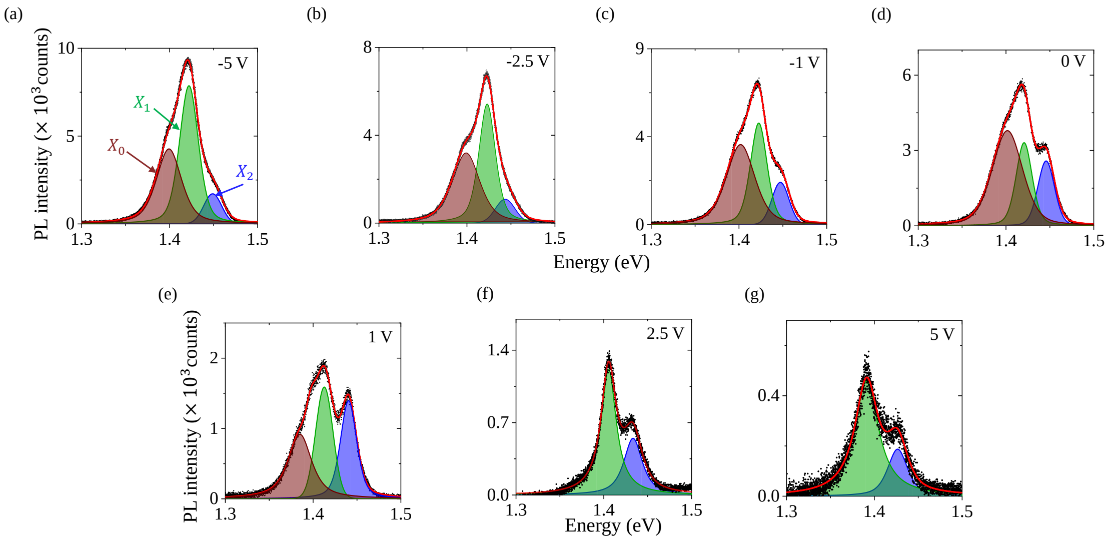

Figure 5: **PL spectra for different gate voltage points.** (a) to (g) represent PL spectra at -5V, -2.5V, -1V, 0V, 1V, 2.5V, and 5V. The three ILE resonances are marked in (a). In the n-doping regime, they evolve gradually, showing quick disappearance of X_0 resonance (after $V_g > 2V$).

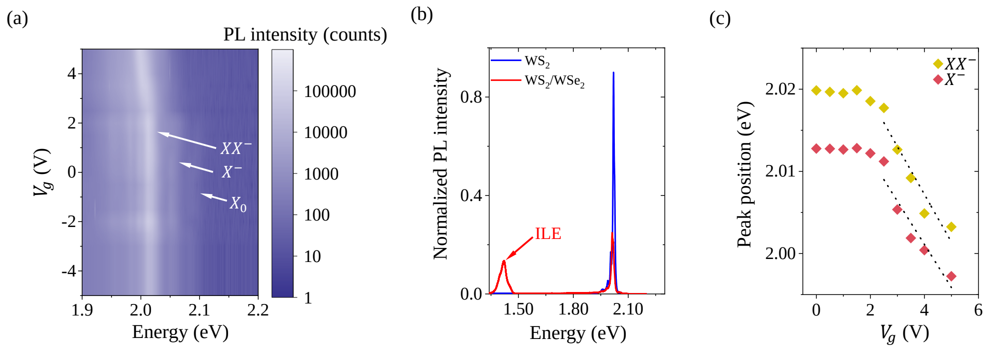

Figure 6: **Trion and biexciton peaks of WS_2 with increasing n-doping.** (a) Color plot of both X^- and XX^- of WS_2 (from the hetrojunction) showing similar redshift as the ILE peaks. (b) A comparison of PL between isolated 1L WS_2 and WS_2/WSe_2 junction is shown. A clear reduction in WS_2 intralayer excitonic emission can be seen due to charge transfer (around 2.02 eV). WSe_2 intralayer excitonic emission is almost completely quenched at the heterojunction. This proves strong interlayer coupling. (c) X^- and XX^- of WS_2 showing similar redshift ($\approx$5 meV/V, dotted black line) as PL peak shift of X_2 ILE with increasing n-doping.

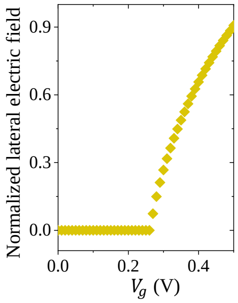

Figure 7: **Local lateral field from Poisson equation.** The calculated local lateral electric field obtained from the Poisson equation as a function of V_g.

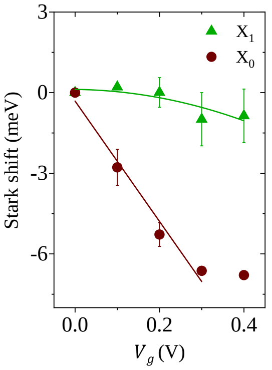

Figure 8: **Stark effect measurement from a different sample.** The Stark effect measurement is repeated in another sample (D4). X_0 and X_1 exhibit linear and parabolic Stark shift, respectively as a function of the gate voltage. This is in good agreement with the results obtained from sample D1 (Figure 2f of main text).

References

[1] Kha Tran, Galan Moody, Fengcheng Wu, Xiaobo Lu, Junho Choi, Kyounghwan Kim, Amritesh Rai, Daniel A Sanchez, Jiamin Quan, Akshay Singh, et al. Evidence for moiré excitons in van der waals heterostructures. Nature, 567(7746):71–75, 2019.

[2] Qinghai Tan, Abdullah Rasmita, Zhaowei Zhang, KS Novoselov, and Wei-bo Gao. Signature of cascade transitions between interlayer excitons in a moiré superlattice. Physical Review Letters, 129(24):247401, 2022.

[3] Fengcheng Wu, Timothy Lovorn, and AH MacDonald. Theory of optical absorption by interlayer excitons in transition metal dichalcogenide heterobilayers. Physical Review B, 97(3):035306, 2018.

[4] Frederik Lohof, Johannes Michl, Alexander Steinhoff, Bo Han, Martin von Helversen, Sefaattin Tongay, Kenji Watanabe, Takashi Taniguchi, Sven Höfling, Stephan Reitzenstein, et al. Confined-state physics and signs of fermionization of moiré excitons in $WSe_2/MoSe_2$ heterobilayers. arXiv preprint arXiv:2302.14489, 2023.
